# Anatomic extension-based description for rotator cuff calcifications: retrospective analysis of 100 consecutive cases

**DOI:** 10.1051/sicotj/2026004

**Published:** 2026-04-20

**Authors:** Daniel Moya, Jorge Rojas Liévano, Diego Gómez, Federico Alfano, Christos Koukos, Daniel Veloz Serrano, Ricardo Vera

**Affiliations:** 1 Department of Orthopaedic Surgery, Buenos Aires British Hospital Buenos Aires Argentina; 2 Department of Orthopaedics and Traumatology, Fundación Santa Fe de Bogotá Bogotá Colombia; 3 Department of Orthopaedic Surgery, Clinica Universidad de Navarra Pamplona Spain; 4 Department of Orthopaedic Surgery, Sports Trauma and Pain Institute Thessaloniki Greece; 5 Department of Orthopaedic Surgery, Centro Médico La Costa Asunción Paraguay

**Keywords:** Calcific tendinopathy, Rotator cuff, Intraosseous migration, Bone erosion, Humeral osteolysis

## Abstract

*Background*: Rotator cuff calcific tendinopathy (RCCT) has traditionally been described as a localized enthesopathy. However, calcium deposits sometimes extend beyond the enthesis into adjacent soft tissues or humeral bone, resulting in atypical patterns not considered in existing classification systems. Failure to recognize these patterns can lead to diagnostic errors or the indication of unnecessary invasive diagnostic procedures. *Methods*: In order to describe atypical patterns and to assess their incidence, 100 consecutive shoulder cases with radiographically confirmed RCCT were retrospectively reviewed. Calcific deposits were categorized by tendon involvement, size, and morphology. Based on imaging findings, deposits were also classified according to their anatomic location and extension into: Type I (enthesis-confined), Type II (extension into soft tissue), and Type III (bone involvement). Associations between patient characteristics, calcification size, morphology, and location were analyzed. *Results*: According to the proposed classification, 67% of cases were Type I, 14% showed soft tissue extension (Type II), and 19% involved bone (Type III). Type III group showed a significantly higher proportion of females (83%) compared to the entire cohort (54%) (*p* < 0.001). Larger deposits (>15 mm) were significantly associated with bone involvement (*p* < 0.01). *Conclusion*: Extension of calcium deposits beyond the rotator cuff enthesis was a frequent finding in this series. Incorporating an anatomic extension-based classification may enhance diagnostic precision, possibly avoiding invasive differential diagnostic procedures. *Level of Evidence*: IV.

## Introduction

Rotator cuff calcific tendinopathy (RCCT) is a common cause of shoulder pain characterized by hydroxyapatite crystal deposition within the rotator cuff tendons [1]. The location of the deposits has traditionally been described as a localized enthesopathy [1]. However, calcium location sometimes extends beyond the enthesis into adjacent soft tissues or humeral bone, resulting in atypical patterns [2–19]. Failure to recognize these patterns may lead to confusion with more aggressive pathologies, such as infection or neoplasm, and lead to the indication of unnecessary invasive diagnostic procedures [1, 3, 5, 7, 14, 16, 19].

The most used classification systems [20, 21] describe the calcium deposits as being limited only to the enthesis and do not include the description of their anatomical extent. The anatomical variants have been exceptionally described in large consecutive case series of rotator cuff calcifications [15], and a classification that encompasses their different location patterns has not been proposed.

The purpose of this study is to analyze the imaging characteristics of calcific deposits in a consecutive series of patients with RCCT, and to propose an anatomy-based approach incorporating the extent of tissue involvement. The secondary objective is to report the relative frequency of the different types of anatomical extension of calcium deposits and to describe variables that may predict the greater likelihood of these findings.

This descriptive framework is intended to improve the detection of under-recognized imaging patterns to aid in differential diagnosis and support clinical decision-making in patients with atypical presentations.

## Material and methods

This retrospective observational study included 100 consecutive shoulders diagnosed with rotator cuff calcific tendinopathy (RCCT) between January 2005 and December 2020 at a single tertiary academic center. Institutional review board approval was obtained, and the requirement for informed consent was waived due to the retrospective design.

Patients with a history of prior shoulder surgery, trauma, or coexisting neoplastic, infectious, or systemic inflammatory conditions affecting the shoulder were excluded.

Eligible patients had radiographic evidence (including anteroposterior and lateral scapula views) of calcific deposits at or near the rotator cuff tendons, with either advanced imaging (MRI and/or CT) and clinical follow-up supporting the diagnosis.

Calcification size was classified using Bosworth’s original system [20]: tiny deposits were defined as barely perceptible, medium deposits were clearly visible but ≤1.5 cm, and large deposits measured <1.5 cm. Morphologic staging was performed using Gärtner’s classification [21], which categorizes deposits as Type I (homogeneous and well-defined), Type II (heterogeneous or partially resorbed), or Type III (poorly defined).

Advanced imaging was used when the characteristics of the radiographic images suggested a pattern other than the conventional one (large sizes according to Bosworth, erosion of the cortex, and/or intraosseous locations). MRI was included in 50 cases, CT scan in 17, and both MRI and CT scan in 10 cases.

One patient had undergone a diagnostic needle bone biopsy to rule out a tumor lesion before being referred to our clinic. This patient had a history of multiple surgeries for breast cancer. The anatomopathological study ruled out the presence of neoplastic cells.

All imaging studies were independently reviewed by two fellowship-trained shoulder surgeons. The reviewers were blinded to clinical data, and discrepancies were resolved by consensus. A pilot analysis of 10 cases was conducted to refine the classification system and ensure consistency prior to formal data collection.

**Figure 1:**
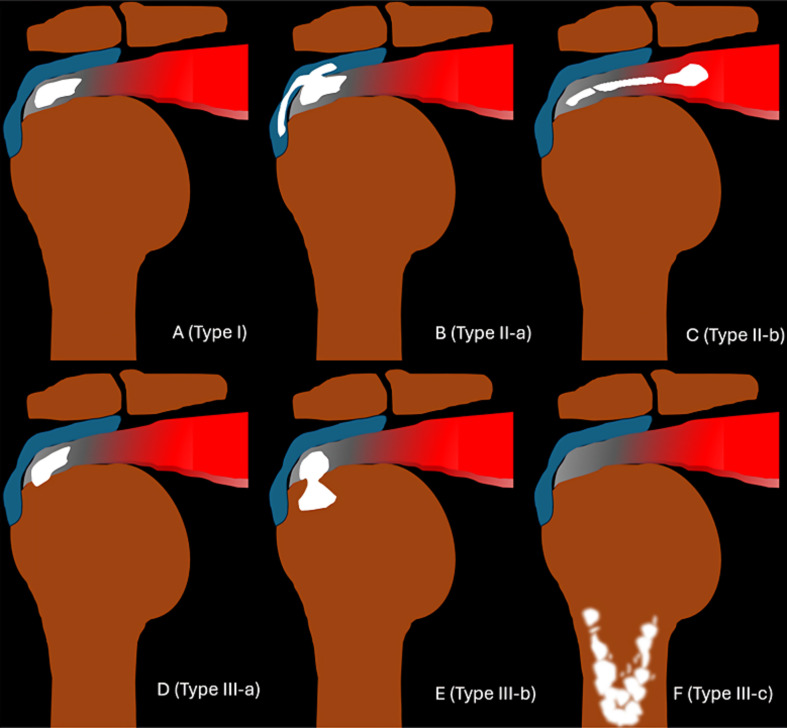
*Schematic illustration of the proposed imaging classification system for rotator cuff calcific tendinopathy.* A. Type I – Enthesis-confined calcification. B. Type II-a – Extension into the subacromial-subdeltoid bursa. C. Type II-b – Medial migration along the myotendinous junction (“comet-tail”). D. Type III-a – Cortical erosion without intraosseous extension. E. Type III-b – Intraosseous extension through a cortical breach. F. Type III-c – Medullary canal involvement (“central progression”).

Calcific deposits were categorized using an imaging-based classification system that describes their anatomic extension (Figure 1). The classification system was developed inductively using a data-driven approach grounded in classification theory.

Type I deposits were confined to the tendon enthesis, with no evidence of spread into adjacent compartments. Type II included deposits that extended into soft tissues beyond the enthesis and were subdivided into Type II-a for extension into the subacromial-subdeltoid bursa, and Type II-b for medial extension toward the myotendinous junction.

Type III referred to bone involvement and was further subdivided into Type III-a, defined as cortical erosion without intraosseous extension; Type III-b, involving intraosseous extension through a cortical breach; and Type III-c, characterized by calcifications entirely embedded within bone, including medullary extension.

All patients were followed clinically until discharge. Of the total patients, 83 had favorable outcomes with conservative treatment, including the application of focused shock waves in 72 of them. Of the remaining 17 patients, four were treated with needle puncture under ultrasound guidance (one of whom subsequently required shock wave therapy), and the rest underwent surgery.

### Statistical analysis

All analyses were conducted using SPSS version 27.0 (IBM Corp., Armonk, NY). Continuous variables were reported as mean ± standard deviation or median with interquartile range, depending on normality, which was assessed using the Shapiro-Wilk test and histogram inspection. Comparisons between groups were performed using chi-square or Fisher’s exact test for categorical variables, and independent *t*-tests or Mann–Whitney U tests for continuous variables, as appropriate. To explore associations between calcification size and bone involvement, patients with Type III lesions were compared to those with Types I and II. A two-tailed *p*-value < 0.05 was considered statistically significant.

## Results

The study included 100 patients with radiographically confirmed RCCT. The mean age of the patients was 53.4 ± 9.3 years (range, 34–73 years), with 54% of the patients being female.

The supraspinatus was the most frequently involved tendon (83%), followed by the infraspinatus (13%), subscapularis (3%), and teres minor (1%).

According to the Bosworth classification [20], 10% of calcifications were tiny, 55% were medium (≤ 15 mm), and 35% were large (>15 mm). Regarding morphology, 58% were Gärtner [21] Type I, 28% Type II, and 14% Type III. These distributions are summarized in Tables 1–3.

**Table 1:** Number and percentage of cases are presented by tendon involved and imaging classification: Type I (enthesis), Type II (soft tissue extension), and Type III (bone involvement, with subtypes III-a, III-b, and III-c).

Tendon	Total	Type I	Type II	Type III (Total)	Type III-a	Type III-b	Type III-c
Supraspinatus	83	55 (66%)	13 (16%)	15 (18%)	11 (13%)	4 (5%)	0 (0%)
Infraspinatus	13	10 (77%)	1 (8%)	2 (15%)	0 (0%)	2 (15%)	0 (0%)
Subscapularis	3	2 (67%)	0 (0%)	1 (33%)	1 (33%)	0 (0%)	0 (0%)
Teres minor	1	0 (0%)	0 (0%)	1 (100%)	0 (0%)	0 (0%)	1 (100%)

**Figure 2:**
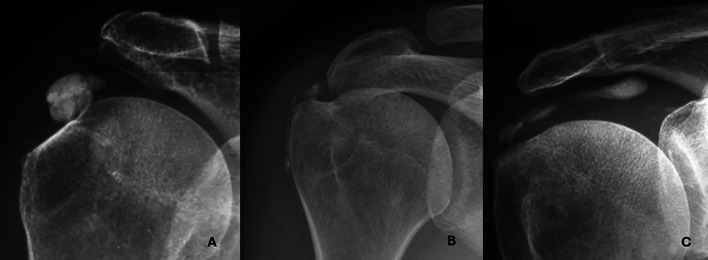
*Representative imaging features of Type I and Type II calcifications.* A. Type I – In situ calcification confined to the tendon, without signs of migration. Note the cortical sclerosis of the greater tuberosity adjacent to the deposit. B. Type II-a – Extension into the subacromial-subdeltoid bursa. C. Type II-b – Medial progression along the supraspinatus tendon, creating a characteristic “comet-tail” appearance.

According to the proposed approach on imaging-defined tissue extension, 67% of calcifications were confined to the enthesis (Type I) (Figure 2A), 14% showed extension into soft tissues (Type II), and 19% involved bone (Type III).

Among Type II lesions, six cases extended into the subacromial-subdeltoid bursa (Type II-a) (Figure 2B), and eight cases extended medially toward the myotendinous junction (Type II-b). The latter subtype exhibited the characteristic “comet-tail” sign, describing a tapering, elongated calcific pattern suggestive of tracking along tendon fibers (Figure 2C).

**Figure 3:**
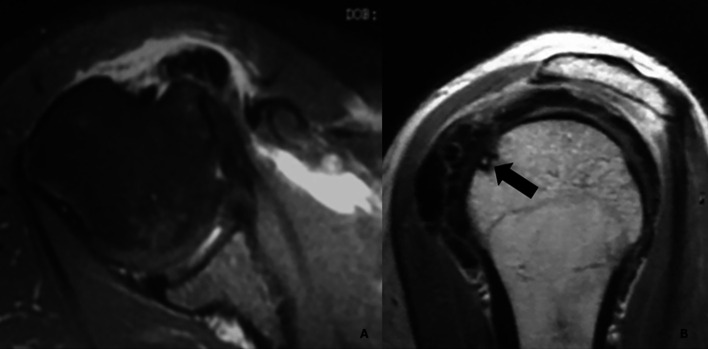
*Magnetic resonance imaging of a Type III-a calcification in the subscapularis tendon.* A. Axial view showing a calcific deposit in direct contact with the cortex of the lesser tuberosity, associated with marked subcoracoid bursitis. B. Oblique sagittal view demonstrating focal cortical erosion at the site of contact (black arrow), without evidence of intraosseous extension.

**Figure 4:**
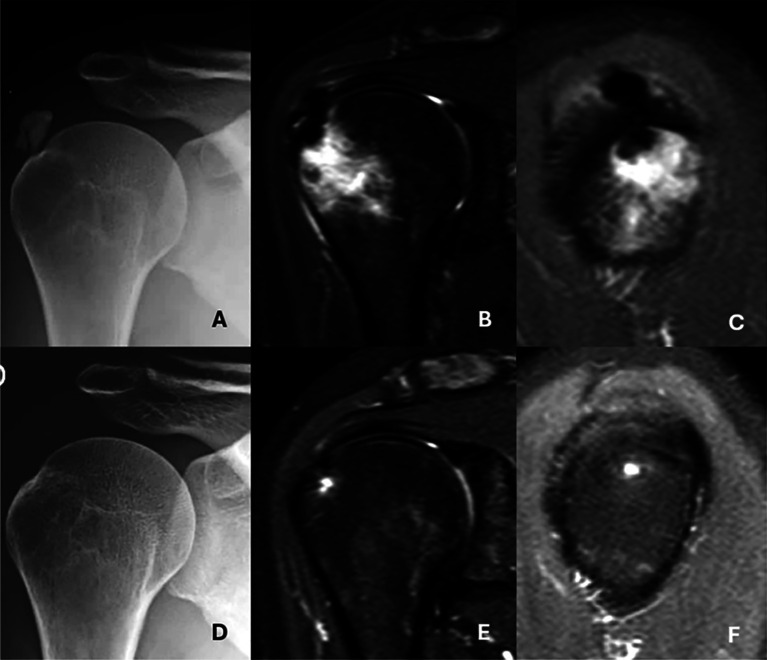
*Advanced intraosseous migration (Type III-b) of a supraspinatus calcification with spontaneous resolution at follow-up.* A. Initial anteroposterior (AP) radiograph showing the calcific deposit. B. Coronal MRI revealing intraosseous extension of the deposit with associated bone marrow edema. C. Oblique sagittal MRI demonstrating the “hourglass sign,” indicating tendon-to-bone continuity. D. Follow-up AP radiograph two years later showing complete disappearance of the calcification. E. Coronal MRI confirming resolution of the intraosseous deposit. F. Oblique sagittal MRI showing complete resolution of both the calcification and bone marrow edema.

**Figure 5:**
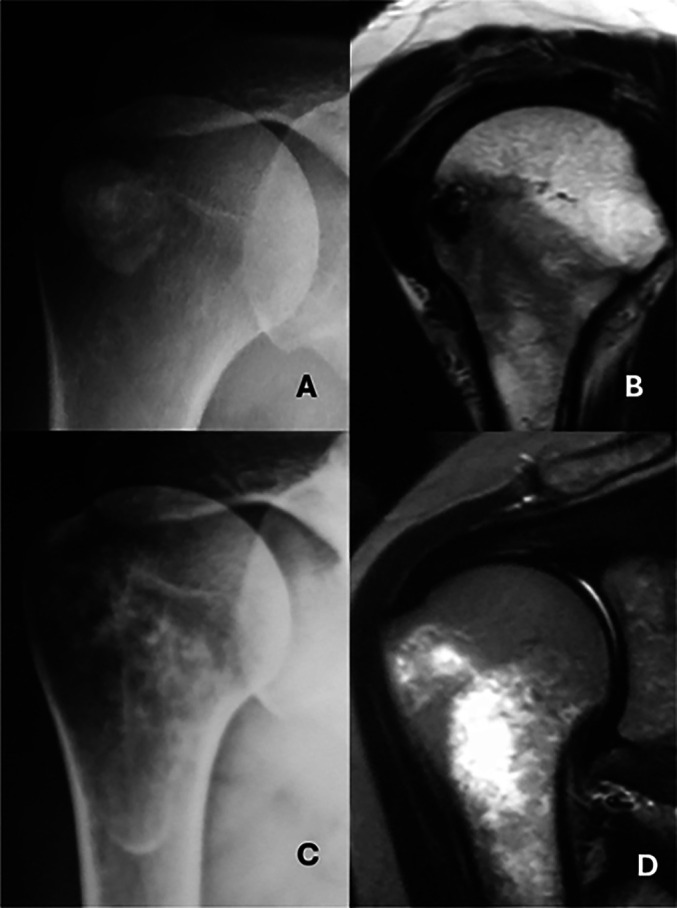
A. AP view showing a calcification located in the tendon of the infraspinatus muscle. B. Oblique sagittal MRI view showing the calcification and the intraosseous deposit. C. Anteroposterior (AP) radiographic image showing extension to the medullary canal of the humerus D. MRI coronal view highlights intramedullary extension.

Of the 19 Type III cases with bone involvement, twelve were classified as Type III-a (cortical erosion without intraosseous extension) (Figure 3), six as Type III-b (intraosseous extension through a cortical breach) (Figure 4), and one as Type III-c (deep medullary involvement) (Figure 5). The “hourglass sign”, indicating continuity between the tendon deposit and intraosseous component, was observed in 83% of Type III-b cases (Figure 4C).

There were no significant differences in age among the three groups studied. Type III group showed a significantly higher proportion of females (83%) compared to the entire cohort (54%) (*p* < 0.001).

Type III involvement occurred in 18% of supraspinatus cases, 15% of infraspinatus cases, and 33% of subscapularis cases. Notably, the only teres minor case presented with central intraosseous extension (Type III-c) (Table 1; Figures 1F and 5). The infraspinatus location showed a stronger association with intraosseous subtypes.

Extension into bone was significantly associated with calcification size. Among large deposits (>15 mm), 26% exhibited bone involvement (Type III), compared to 15% of medium-sized deposits (5–15 mm) and only 2% of tiny deposits (<5 mm). This trend was statistically significant (*p* < 0.01), indicating that larger deposits are more likely to extend into bone (Table 2). Types III-b and III-c lesions were almost exclusively associated with medium and large deposits.

**Table 2:** Distribution of calcification sizes (Bosworth classification) by imaging type. Number and percentage of cases are shown by size category: small (<5 mm), medium (5–20 mm), and large (>20 mm), across classification types. Larger deposits were more frequently associated with bone involvement (Type III).

Size	Total	Type I	Type II	Type III (Total)	Type III-a	Type III-b	Type III-c
Tiny	10	8 (80%)	0 (0%)	2 (20%)	2 (20%)	0 (0%)	0 (0%)
Medium ≤ 15 mm	55	44 (80%)	3 (5%)	8 (15%)	7 (13%)	1 (2%)	0 (0%)
Large (>15 mm)	35	15 (43%)	11 (31%)	9 (26%)	3 (9%)	5 (14%)	1 (3%)

Calcification morphology, as defined by the Gärtner system [21], did not reliably predict extension pattern. Well-defined calcifications (Gärtner Type I) exhibited invasive anatomical behavior in 12% of the cases as did 36% of heterogeneous (Gärtner Type II) and 14% in poorly defined deposits (Gärtner Type III) (Table 3).

**Table 3:** Distribution of Gärtner stages by imaging classification type. Number and percentage of cases are presented by Gärtner stage: Type I (homogeneous), Type II (heterogeneous), and Type III (fragmented), across imaging classification types. Bone involvement (Type III) was observed across all Gärtner stages.

Gärtner Type	Total	Type I	Type II	Type III (Total)	Type III-a	Type III-b	Type III-c
Type I (Homogeneous)	58	46 (79%)	5 (9%)	7 (12%)	3 (5%)	3 (5%)	1 (2%)
Type II (Heterogeneous)	28	12 (43%)	6 (21%)	10 (36%)	8 (29%)	2 (7%)	0 (0%)
Type III (Fragmented)	14	9 (64%)	3 (21%)	2 (14%)	1 (7%)	1 (7%)	0 (0%)

## Discussion

Rotator cuff calcific tendinopathy (RCCT) is traditionally regarded as a benign and self-limiting condition, commonly restricted to the tendon enthesis and dominated by deposits in the supraspinatus [1]. However, the results of this study challenge this conventional understanding. In this cohort of 100 consecutive RCCT cases, only 67% of calcifications were confined to the enthesis (Type I). A considerable proportion extended beyond the tendon into adjacent soft tissues (14%) or into the bone (19%).

An extension-based classification approach integrates key features of deposit location, directional extension, and osseous involvement to capture the diverse imaging phenotypes of RCCT.

Despite the novelty and clinical relevance of this classification, several limitations must be acknowledged. First, the retrospective design and non-standardized imaging protocols may have introduced selection bias. While advanced imaging was available in most cases (MRI and/or CT in 77%), subtle extension patterns may have been underdetected in radiograph-only cases. Second, the interobserver reliability of the classification was not formally tested and should be a focus for future validation studies. However, unlike existing frameworks such as the Bosworth [20] size or Gärtner [21] staging classification – both of which are based solely on radiographic dimensions or morphology – the anatomic approach includes distribution and cross-sectional imaging.

Several prior reports have noted atypical extensions of calcific deposits, including intrabursal and intraosseous presentations [2–19], but these are often reported in isolated case series without a unifying classification (Table 4). Malghem et al. [2] described cortical erosions and medullary diffusion; Pereira et al. [3] noted intramuscular spread through tendon tears. Hutchinson et al. [11] and Zampa et al. [19] highlighted the diagnostic confusion that intraosseous cases may cause when not recognized as a consequence of RCCT.

**Table 4:** Case reports and series that include progression of rotator cuff calcifications to soft tissues and bone structures.

First author	Cases	Location of the calcification
Pereira [3]	11	Intramuscular migration
Seyahi [4]	5	Osseous involvement of the humeral head
Chan [5]	1	Osseous involvement of the humeral head
Chagnaud [6]	1	Osseous involvement of the humeral head
Della Valle [7]	1	Subacromial–subdeltoid bursa & Osseous involvement of the humeral head
Flemming [8]	50	Osseous involvement in different areas of the skeleton
Gwalani [9]	1	Osseous involvement of the humeral head
Jain [10]	1	Osseous involvement of the humeral head
Hutchinson [11]	1	Osseous involvement of the humeral head
Jain [12]	1	Osseous involvement of the humeral head
Martin [13]	1	Osseous involvement of the humeral head
Nogueira-Barbosa [14]	7	Osseous involvement of the humeral head
Porcellini [15]	43	Osseous involvement of the humeral head

The findings in this study consolidate these disparate observations into a reproducible system that emphasizes the anatomic extension, a point insufficiently emphasized in prior literature.

Bone involvement was observed across all Gärtner stages, including 12% of deposits with homogeneous morphology (Gärtner Type I), reinforcing that early radiographic appearance does not reliably predict the extent of anatomic extension. Similarly, while larger deposits were statistically more frequently associated with bone involvement, 15% of medium-sized and even 2% of tiny deposits showed cortical or intraosseous extension. Thus, deposit size alone, as in the Bosworth classification [20], is also an insufficient predictor of behavior.

It is considered that, ideally, a classification should also address treatment and prognosis. This is difficult to achieve in a condition like rotator cuff calcifications, where many variables must be considered. Decision-making must include the patient’s symptoms, the size of the deposit, its location, the stage of progression, and the response to previous treatments. In fact, none of the classifications currently in use incorporates all of these variables.

The anatomic extension-based classification has diagnostic value. It may help prevent diagnostic errors. Type III lesions, particularly those with intraosseous extension (Type III-b or III-c), may mimic bone tumors, osteomyelitis, or other aggressive pathologies on MRI, especially when bone marrow edema is present [3, 11, 19]. Awareness of the “hourglass” sign and the absence of destructive features can help differentiate RCCT from malignant or infectious processes, reducing unnecessary biopsy or overtreatment.

A predisposition to intraosseous migration related to the patient’s gender had not been previously recognized. In our series, there was a higher incidence of females (83%) in cases of bone injury compared to the general incidence of rotator cuff calcifications (54%). This higher frequency in women could be related to lower bone density, as it is well known that the mineral density of the greater tuberosity is affected by general osteoporosis [22].

From a therapeutic standpoint, the identification of soft tissue or bone extension may have implications [16, 23–25]. Extension of the deposit medially beyond the acromioclavicular joint (Type II-b) has been considered a poor prognostic factor for conservative treatment [23, 25]. Intrabursal extension (Type II-a) may respond better to lavage or aspiration, while intraosseous lesions may be less amenable to percutaneous procedures and may require surgical management [25]. However, spontaneous radiographic and clinical resolution was common in this group, as seen in Figures 4D–4F in a patient followed over two years – highlighting that intraosseous extension does not always imply aggressive or refractory disease, a point also noted by Hutchinson et al. [11]. It has been hypothesized that calcific migration from tendon to bone occurs in an advanced evolutionary phase involving resorption [14]. For these reasons, it is advisable to exhaust conservative treatment options in these cases.

Finally, while intraosseous and intramuscular extensions are frequently described in isolated case reports, this study represents one of the first attempts to systematically integrate these findings into a unified and reproducible imaging classification. This framework, if validated in prospective cohorts, may serve as a practical tool for radiologists, orthopedic surgeons, and sports medicine clinicians managing patients with RCCT.

## Conclusion

The proposed anatomic extension-based classification system provides a descriptive framework to capture a broad range of RCCT patterns, incorporating novel imaging signs and subtypes. These findings offer a foundation for improved recognition and future studies on clinical relevance.

## Data Availability

This article has no associated data generated.
